# In Silico Insights of L-Glutamate: Structural Features in Vacuum and in Complex with Its Receptor

**DOI:** 10.1155/2013/872058

**Published:** 2013-11-06

**Authors:** Janneth Gonzalez, George E. Barreto

**Affiliations:** Departamento de Nutrición y Bioquímica, Facultad de Ciencias, Pontificia Universidad Javeriana, Bogotá D.C., Colombia

## Abstract

Structural properties of the glutamate in vacuum and in complex with its receptor were analyzed. The analysis was focused on global properties, attempting to characterize features such as overall flexibility and common trends in the conformation set. The glutamate, as other ligands in complex with the receptor, adopts a spatial conformation that corresponds to one of the possible molecular equilibrium states in physiological conditions. The glutamate forms an extended structure for all cases, but the energy of the glutamate round out form is lower than the extended glutamate form. The results showed the glutamate as a flexible molecule, which can easily adapt to different interacting environments, and it can be considered as an approximation to address why glutamate interacts with a great number of molecules.

## 1. Introduction

The amino acid L-glutamate is considered the main excitatory neurotransmitter in mammals Central Nervous System (CNS) [1, 2]. Most of the CNS synapses use glutamate as a fast neurotransmitter, and at least 60% of the synapses in the human brain are glutamatergic [3]. The glutamate has an important physiological role in many aspects of the normal brain function such as cognition, memory, learning, nervous system development, cellular migration, cellular differentiation, and neural death [4, 5]. After being released from a presynaptic cell, glutamate diffuses across the synaptic cleft and binds to its specific receptors in the cell membrane of a postsynaptic cell. Once across the synaptic cleft, glutamate is recognized by glutamate receptors from a high variety of other molecules. Although the mechanism of molecular recognition has long been considered in a key-keyhole relationship, short-range forces have been considered as the primary cause of such interactions [6].

The glutamate (L-isomer) causes depolarization and excitation of neurons, but it does so by acting on a variety of receptors. The AMPA/kainate receptors respond to the glutamic acid analogues, *α*-amino-3-hydroxy-5-methylisoxazole-4-propionic acid (AMPA) and kainic acid. Other receptors, N-methyl-D-aspartate (NMDA) receptor, also belong to the ion-channel-linked superfamily, and there is a population of metabotropic receptors, as upon activation they simulate a second messenger transduction system [7].

The behavior of different neurotransmitters has been mainly studied using physiological and pharmacological techniques in both vertebrate and invertebrate brains, and recent techniques in molecular biology have clarified the amino acid sequences of their binding sites [8–16]. However, it is important to note that natural and synthesized analogues with small differences in their chemical structures exert completely different influences and could even have opposite ones. This fact gives rise to an important question: which structural and chemical characteristics are necessary for the ligand to recognize its receptor? The accepted ideas about the molecular recognition pattern are that it occurs because a ligand and its receptor are geared toward each other in a relationship resembling that between a key and a keyhole, so their geometrical structures are determinant.

The glutamate, as other ligands in complex with the receptor, adopts a spatial conformation or tridimensional structure that corresponds to one of the possible molecular equilibrium states in physiological conditions. The conformations adopted by glutamate in complex with the receptor are the result of ligand-receptor interactions at an atomic level. It is important to consider that the allowed conformations for a molecule could have dramatic effects on the molecule activity or reactivity. Therefore, it is necessary to consider all the possible conformations for molecular properties studies.

To assess the structural properties of the glutamate, we analyzed a conformational ensemble. The analysis was focused on global properties, attempting to characterize features such as overall flexibility and common trends in the conformation set. It is important to have in mind that the different conformational analysis can be applied to any collection of molecular conformations. These may be generated by sampling techniques with theoretical structures but can also have an experimental origin, such as NMR models or different X-ray structures of the same molecule (or analogous molecules). In the present study, we calculated the structural conformations of glutamate (a) in complex with receptor and (b) in vacuum form, as an approach for understanding the mechanism of the recognition of a ligand by its receptor.

## 2. Materials and Methods

### 2.1. Experimental Structures

For this study, we used 22 structures of glutamate receptor monomers from different subunits solved by X-ray crystallography and deposited in PDB [17]. The 22 structures were derived from 7 crystals, which 5 of them belong to ionotropic receptors and 2 belong to metabotropic receptors (Table 1).

To estimate the uncertainty grade in the atomic positions from the selected glutamate structures, we measured the following internal variables for the heavy atoms: (a) bonds length; (b) bond angles; and (c) dihedral angles. The statistical analyses were made with program Statistica v.6.0 (v. 5.5). To make a comparison among structures, we performed a multiple structural alignment with the software VMD for molecular analysis [18].

### 2.2. Theoretical Structures

**Figure 1:**
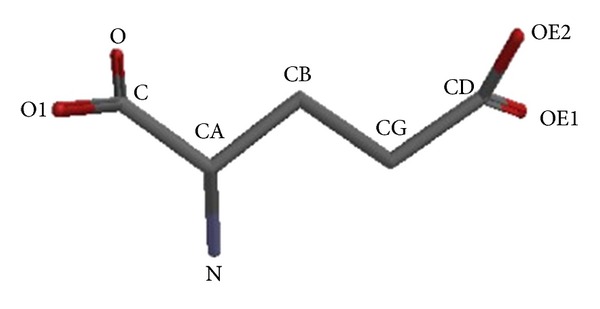
Chemical structure of glutamate. The side chain exists in its negatively charged deprotonated carboxylate form at pHs greater than 4.1; therefore, it is also negatively charged at physiological pH ranging from 7.35 to 7.45.

Under neutral pH in solvated conditions, the carboxyl groups and amino groups in glutamate are deprotonated and protonated, respectively. We thus used the ionized models and calculated them in vacuum to obtain the best structural conformation of glutamate. The initial conformation of glutamate was built and optimized with MOE (v. 2007-11) using standard values for the internal 0 variables. The standard nomenclature use by PDB was considered to point out the heavy atoms in the glutamate molecule (Figure 1).

**Figure 2:**
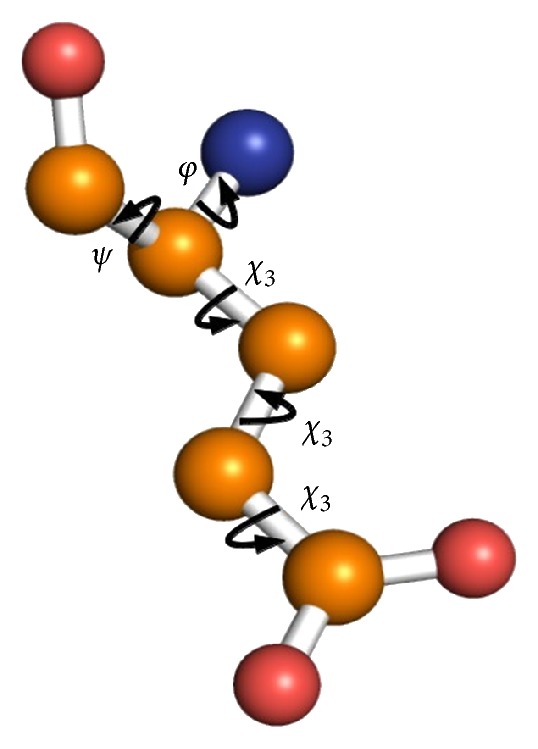
Dihedral angles in glutamate. The angles show the main degrees of freedom for the backbone (*φ* and *ψ* angles) and the side chain (*χ* angles) of glutamate. The figure shows a ball-and-stick representation of glutamate, which has three *χ* angles. The fading conformations in the background illustrate a rotation around *χ*_1_.

It is important to note that the number of tridimensional structures expands geometrically with the number of possible combinations for the dihedral angles in the structure. Considering that the conformers differ primarily in their torsion angles, in this study we focused on the conformational space. The exploration of the conformational space was made by changing the dihedral angles in the 30 degrees step (Figure 2).

The database of glutamate conformers used in this study was based on minimal energy glutamate conformations, elimination of duplicated structures, and those with an atomic RMSD value higher than 0.1 Å. As a final result, 10 stable structures of glutamate were found. These structures were optimized again by GAMMES program (GAMMES v. Sep 7/2006 (R4)) using RHF/6-31G** approach. Geometry optimization was further performed for the most stable structure of each molecule, and the frequency was then calculated to confirm that the optimized structure was the ground state geometry, using the 6-31G** basis set.

## 3. Results

### 3.1. Experimental Structures

It is often assumed that crystal structures have to be obtained at sufficiently high resolution in order to perform macromolecular refinement, and what are considered “acceptable” have been pushed to lower diffraction resolutions. In most cases, the crystal quality is measured according to the resolution value. Although in this study we used two crystals resolved with low resolution, namely, 2CMO (2.65 Å) and 1ISR (4.00 Å), we found no difference when these structures were compared with the high-resolution structures. 

The results show that the average of bond lengths values is not statistically different neither between monomers, which come from the same crystal, nor for monomers coming from different crystals. This result shows that at least for the studied structures, the bond length can be viewed at random, and they do not respond to the fact of coming from different crystals or the resolution degree. According to these results, all structures obtained may be used as models for the following analysis.  Table 2 shows the descriptive statistics for bond length variable (*n* = 22).

The variation coefficient is less than 1.00%, and this is consistent with the fact that the maximum range reported for the CG-CD bonds with a value that corresponds to 3.86% of the all bonds values average [17]. On the other hand, the similarity among bond angles values does not correspond, due to the fact that monomers come from different crystal or experimental resolutions. It can be considered for this variable that all the chosen structures belong to one population with close variations values (Table 3).

The variation coefficients higher than 1.00% are the maximum values in which the CA-C-O angles have an average value of 6,554°. The degree of similarity observed for the internal variables discussed previously (bond lengths and bond angles) was not observed for the dihedral angles. These differences are mainly due to the different conformations that glutamate can take in complex with its respective original receptor nearly 22 cases analyzed. This result is a qualitative indicator that the glutamate is a dynamic molecule that shows structural heterogeneity with discrete conformational states that share energetic values in close ranges.

**Figure 3:**
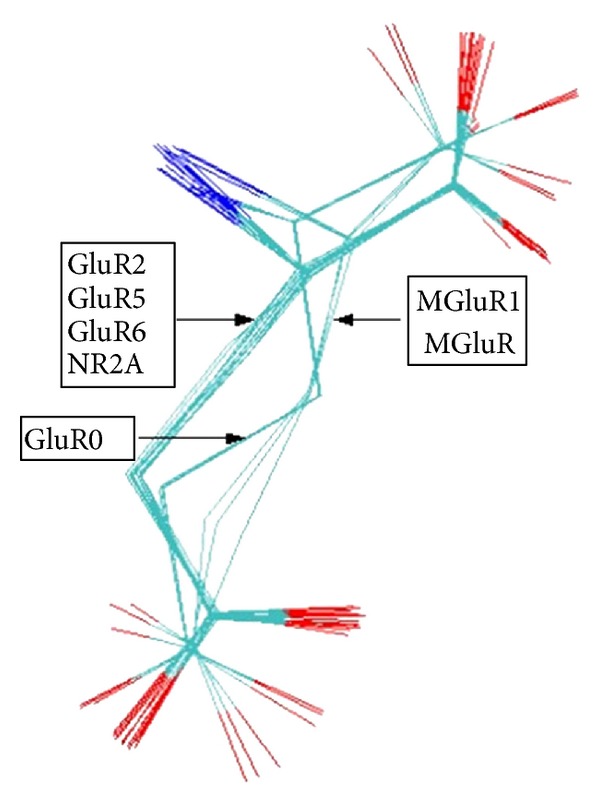
The highest scoring alignment of glutamate. The clusters formed by different conformations are correctly aligned and emerged as a result of specific environments of the binding sites interaction with each of the receptors.

The RMSD value was selected as a quantitative indicator of the structures variability for alternative conformation within the same molecule. The visual analysis of multiple structural alignments allows the identification of three major clusters. These conformations emerged as a result of specific environments of the binding sites interaction with each of the receptors (Figure 3).

The first and second groups are composed of 16 and 3 isolated molecules from ionotropic glutamate receptors crystals, and the third group is composed of 3 isolated molecules from metabotropic glutamate receptors crystals. The distribution of clusters based on the RMSD is shown in Table 4.

**Figure 4:**
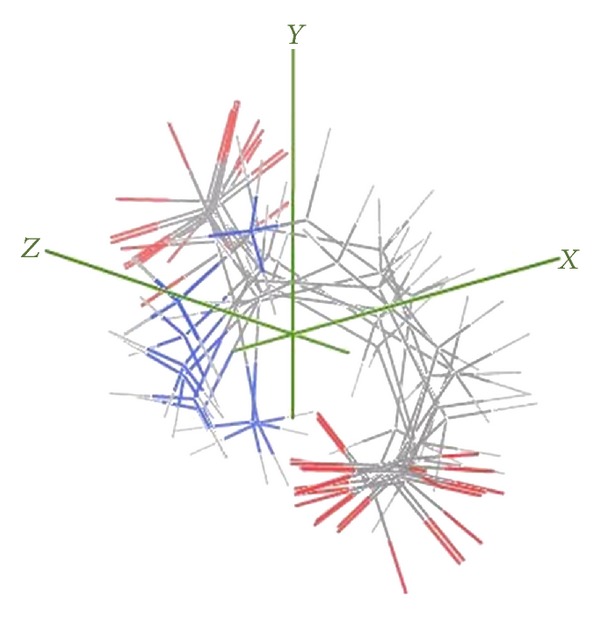
Differences between structural alignments. A backbone overlap according to the same alignment is shown; the backbone RMSD is ≤0.5. The figure shows that the conformations adopted by the glutamate are most stable when rounded out.

After conformational analysis, 22 refined structures were grouped into three clusters based on pairwise main-chain RMSD (Figure 3). Structures in the first cluster (cluster 1) represent > two-thirds of the sample with an RMSD of 0.3 Å RMSD from its center. The second group (clusters 2 and 3) has an RMSD of 2.0 Å from the first cluster center. A structural comparison between minimum energy structures of the three clusters is shown in Figure 4. Although it is hard to distinguish clusters by the way that the experimental restraints are satisfied (i.e., by RMSD deviations; see Table 3), we note that the 16 lowest energy structures as evaluated by the MOE scoring function all belong to cluster 1 (structural statistics are given in Table 3).

### 3.2. Theoretical Structures

The database obtained included 118 structures, which represent locally stable glutamate conformations; neither bond lengths nor bond angles are statistically different, and the values are in a close range (0.001 Å to 0.02 Å) for the bond angles of the 118 glutamate conformers. The results are shown in Table 5.

From the comparison of the dihedral angles from the 118 selected structures, we identified 10 groups of conformations with high structural similarity. These 10 groups were found based on different arrangements for multiple structural alignments (RMSD among groups ≥ 1.552). 

The conformations included in each group share structural similarity (RMSD ≤ 0.5), and the graphic representation of the structural alignments between selected conformations is shown in Figure 4.

The minimum energy conformations adopted by the glutamate molecule show that the amino acid is most stable when rounded out, despite the extended form that it adopts when interacting with the receptor. This extended conformation is adopted because the lateral chain is bonded to water molecules through hydrogen bonding.

**Figure 5:**
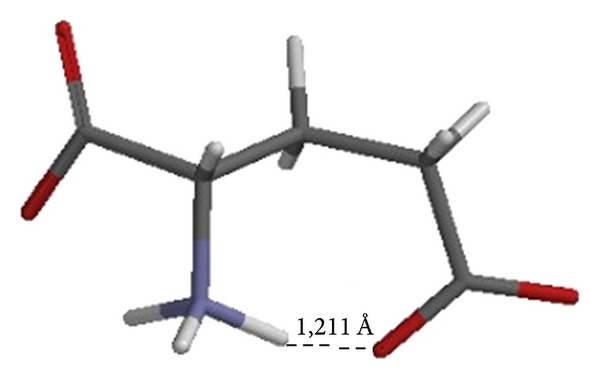
Representation of the glutamate in vacuo shows the propensity to form a hydrogen bond among the amino and carboxyl groups of the principal and side chain, respectively.

Since the ionized glutamate model in free state is not subjected to interactions with surrounded molecules, it is hypothesized that the rounded form adopted is due to the attraction between the amino and carboxyl side chain groups(Figure 5); the attraction and charge compensation between groups in the same molecule generate a propensity to form a hydrogen bond between these two groups (distance H-O 1.211 Å).

**Figure 6:**
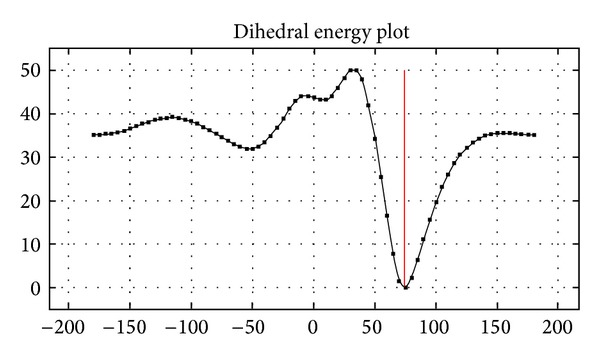
Plot of energy versus dihedrals for the selected glutamate conformers. The glutamate structure in free form is determined for the dihedrals angles combinations for which the molecule energy has the lowest values.

According to the study of the preferential glutamate conformations, it is observed that the maximal variability in the relative position of the heavy atoms is found in the side chain. Figure 6 shows the relationship among the variables energy and dihedrals. The glutamate structure in free form is determined for the dihedrals angles combinations for which the molecule energy has the lowest values.

Although all the conformers obtained share energetic values in a close range (−82.401 y−82,397 Hartrees), the local minimum differs in 1.5 Å (RMSD) average among structures.

## 4. Discussion

The analyses of the classical internal variables of the 22 conformers of glutamate allowed us to identify different groups of conformations according to the type of receptor, which can lead to the idea of the ligand conformation as an indicator of the binding site molecular environment. These results showed the glutamate as a flexible molecule, which can easily adapt to different interacting environments, and it can be considered as an approximation to address why glutamate interacts with a great number of molecules [1–6].

The comparison through the standard method of multiple structures alignment showed that the RMSD values were not dependent on the crystal resolution degree of the diffracted structures. This may be correlated with the standard error of the positional atomic parameters, which can increase if the resolution decreases. Considering that it is not possible to use reliable indicators for the positional standard errors in molecular structures, the use of the RMSD values to compare molecular structures must be considered [19].

The atomic fluctuations of the different conformational states and the structural variability were inherent conditions due to the binding site molecular environment. The molecule flexibility plays a key role in its function [20–23]. Since the RMSD values of the groups obtained are closely related to each other, it was possible to use a representative structural model for each group in order to establish relationships type structural between them for subsequent studies.

The analyses of the RMSD value, according to the glutamate atomic position changes, showed that the variability increased with the atomic distance from the CA. Some hypotheses can be generated from our study: (1) the movement of the principal chain is more conservative in comparison with the side chain if molecule flexibility is considered; (2) the atomic positions of the side chain are variables as the RMSD values show. According to glutamate structure in complex with the receptor binding site reported by Tsuchiya et al. [24] and validated with different subunits of the receptor [8–16], it was found that the glutamate forms an extended structure for all cases, suggesting that there is no number of possibilities to find different geometries when its structure is resolved by X-ray crystallography. The extended form (Figure 3) is different to the round-out form (Figure 4). The energy of the glutamate round out form is lower than the extended glutamate form.

## 5. Conclusion

Glutamic acid is the key amino acid in living organisms. In order to characterize features such as overall flexibility and common trends in the conformation of glutamate in vacuum and in complex with its receptor, we have undertaken theoretical studies. The results showed the glutamate as a flexible molecule, which can easily adapt to different interacting environments, and it can be considered as an approximation to address why glutamate interacts with a great number of molecules; also our results might provide a possible approach for understanding the mechanism of the recognition of glutamate by its receptor.

## Figures and Tables

**Table 1:** Selected glutamate receptors solved by X-ray crystallography.

Subunit	PDB ID	Number of monomers	Seq. length (aa)	Res. Å	Organism	Expression system	Gene
GluR2	1FTJ^13^	3	258	1.90	*Rattus norvegicus *	*Escherichia coli *	Gria2
	2CMO^1^	2	257	2.65	*Rattus norvegicus*	*Escherichia coli*	Gria2

GluR0	1II5^15^	1	221	1.60	*Synechocystis* sp.	*Escherichia coli*	sll1147
	1US4^16^	1	298	1.75	*Thermus thermophilus *	*Escherichia coli*	Prot.
	1US5^16^	1	298	1.50	*Thermus thermophilus*	*Escherichia coli*	Prot.

GluR5	1TXF^17^	1	248	2.10	*Rattus norvegicus*	*Escherichia coli*	Grik1
	1YCJ^18^	2	251	1.95	*Rattus norvegicus*	*Escherichia coli*	Grik1
	2F36^19^	4	254	2.11	*Rattus norvegicus*	*Escherichia coli*	Grik1

GluR6	1S7Y^20^	2	251	1.75	*Rattus norvegicus*	*Escherichia coli*	Grik2
	1S50^21^	1	258	1.65	*Rattus norvegicus*	*Escherichia coli*	Grik2

MGLUR1	1EWK^2^	2	448	2.20	*Rattus norvegicus*	*Autographa *	Mglur1

MGLUR1	1ISR^24^	1	448	4.00	*Rattus norvegicus*	*Spodoptera frugiperda *	Mglur1

**Table 2:** Summary of descriptive statistic for bond length between heavy atoms of glutamate conformers. Variation coefficient is in percentages. The values presented in the table come from monomers from different crystal and different experimental resolutions.

Bond	Average	SD	Min.	Max.	VC (%)
CB-CG	1,523	0,011	1,500	1,544	0,754
CG-CD	1,525	0,013	1,509	1,568	0,873
CD-OE1	1,250	0,009	1,231	1,271	0,684
CB-CA	1,535	0,010	1,524	1,576	0,671
CA-C	1,524	0,008	1,502	1,540	0,504
C-O	1,245	0,010	1,213	1,261	0,833
CA-N	1,491	0,010	1,460	1,514	0,656
C-O1	1,247	0,006	1,237	1,257	0,477
CD-OE1	1,254	0,010	1,242	1,282	0,778

**Table 3:** Summary of descriptive statistic for bond angle between heavy atoms of glutamate conformers. Variation coefficient is in percentages. The values represent monomers from different crystal and different experimental resolutions.

Angle	Average	SD	Min.	Max.	VC (%)
CB-CG-CD	112,999	0,970	111,085	115,479	0,858
CG-CD-OE1	118,545	1,146	114,553	120,904	0,966
CG-CB-CA	114,447	0,984	112,796	116,309	0,860
CB-CA-C	110,027	0,813	108,911	112,036	0,739
CA-C-O	118,796	1,044	117,595	121,377	0,878
N-CA-C	111,063	1,478	108,364	114,181	1,331
CA-C-O1	118,565	1,558	117,090	123,644	1,314
CG-CD-OE2	119,017	1,402	117,371	123,327	1,178

**Table 4:** ID of molecules and receptors from clusters based on pairwise main-chain RMSD. Structures in the first cluster (group 1) represent > two-thirds of the sample with a RMSD of 0.3 Å RMSD. The second cluster (groups 2 and 3) has a RMSD of 2.0 Å.

Group	Molecule (ID)	Receptor number
1	1FTJ-274	GluR2
	1FTJ-275	
	1FTJ-276	
	2CMO-275	
	2CMO-274	
	1TXF-111	
	1YCJ-998	
	1YCJ-999	
	2F36-501	
	2F36-502	
	2F36-503	
	2F36-504	
	1S50-999	
	1S7Y-552	
	1S7Y-551	
	2A5S-1001	

2	1US4-131	GluR0
	1US5-1313	
	1II5-999	

3	1EWK-701	MGluR1
	1EWK-702	
	1ISR-1001	MGluR

**Table 5:** The minimum and maximum values for bond angles of the 118  glutamate conformers. The values are in the range from 0.001 Å to 0.02 Å.

	Min.	Max.	Average
C-CA	1,551	1,555	1,552
CA-N	1,498	1,504	1,501
CA-CB	1,531	1,546	1,538
CB-CG	1,533	1,537	1,535
CG-CD	1,528	1,533	1,530
C-O1	1,253	1,279	1,266
C-O2	1,253	1,279	1,266
CD-OE1	1,254	1,280	1,267
CD-OE2	1,254	1,280	1,267
